# Patient perceptions of oral health care following stroke: a qualitative study

**DOI:** 10.1186/s12903-021-01501-7

**Published:** 2021-03-17

**Authors:** Shilpi Ajwani, Caleb Ferguson, Ariana C. Kong, Amy R. Villarosa, Ajesh George

**Affiliations:** 1 Oral Health Promotion and Oral Health Research, Sydney Local Health District Oral Health Services/Sydney Dental Hospital/University of Sydney, Sydney, 2010 Australia; 2 Heart Foundation Postdoctoral Fellow, Western Sydney Nursing and Midwifery Research Centre, Western Sydney University/Western Sydney Local Health District/Centre for Oral Health Outcomes and Research Translation (COHORT)/Ingham Institute for Applied Medical Research/Translational Health Research Institute (THRI) Blacktown Clinical and Research School Blacktown Hospital, Marcel Crescent, Blacktown, NSW 2148 Australia; 3 Centre for Oral Health Outcomes and Research Translation (COHORT), South Western Sydney Local Health District/Ingham Institute for Applied Medical Research, Western Sydney University, Liverpool, NSW 2170 Australia; 4 Centre for Oral Health Outcomes and Research Translation (COHORT), South Western Sydney Local Health District/Ingham Institute for Applied Medical Research, Western Sydney University, Liverpool, NSW 2170 Australia; 5 Centre for Oral Health Outcomes and Research Translation (COHORT), South Western Sydney Local Health District/Ingham Institute for Applied Medical Research/Translational Health Research Institute/University of Sydney, Western Sydney University, Liverpool, NSW 2170 Australia

**Keywords:** Stroke, Oral health, Delivery of Health Care, Integrated, Qualitative research

## Abstract

**Background:**

Stroke is a serious cerebrovascular disease and is one of the world’s leading causes of disability. Maintaining good oral health is a challenge among those hospitalised after stroke. A multidisciplinary approach to oral care involving non-dental professionals can be beneficial in improving oral health outcomes for patients. The aim of this study was to understand the perceptions of stroke survivors regarding oral healthcare across acute and rehabilitation settings.

**Methods:**

A descriptive qualitative approach was used. Face-to-face semi-structured interviews were conducted. A framework analysis was employed to analyse the data. Patients who had recently experienced a stroke were purposively recruited across both acute and rehabilitation settings, at two metropolitan hospitals in Sydney, Australia. In total, 11 patients were interviewed.

**Results:**

Although participants recognised the importance of oral health, few understood the link between oral and general health. Regular oral hygiene practices varied since having stroke, with a few receiving oral care assistance from nurses. Time, cost and lack of information were some barriers to accessing dental services, while supportive measures such as coordination of oral care, financial subsidy and nurse assistance were strategies proposed to support oral care practices amongst stroke survivors.

**Conclusions:**

There is scope to improve current models of oral care in stroke. While stroke survivors understand the importance of oral care, an integrated oral health model with a multidisciplinary approach could improve health outcomes.

**Supplementary Information:**

The online version contains supplementary material available at 10.1186/s12903-021-01501-7.

## Background

Impacting 1 in 4 people worldwide [1], stroke is one of the leading causes of death and loss in disability-adjusted life-years resulting in significant social and economic burden in Australia and across the world [2]. An estimated two-thirds of Australian people are unable to perform activities of daily living unassisted following a stroke [3]. Stroke survivors may also have deficits relating to movement, communication, cognitive issues, pain and depression [4, 5]. As health professionals are often focused on stabilising their acute medical condition, an often overlooked area of need among stroke survivors is oral health [6].

Stroke significantly impacts on a person’s ability to self-care, and increases their reliance on others to support activities of daily living, including their oral hygiene. Up to 75% of patients may be unable to brush their teeth or maintain their oral health due to impaired cognitive and physical abilities [7]. Motor dysfunction, a typical feature of stroke, can contribute to dysphagia. Along with poor oral hygiene, this greatly increases the risk of aspiration pneumonia [8–12]. Aspiration pneumonia is a life-threatening, acute infection which develops after a large volume of aspiration is accumulated in the lungs. This aspiration often transports a significant bacterial load from the oral cavity or the upper gastrointestinal tract into the lungs, resulting in an infection that can deteriorate into pneumonia [13, 14]. Meta-analyses demonstrate that including oral care with chlorhexidine as part of a comprehensive bundle can reduce ventilated aspiration pneumonia by 10–30% in cardiac surgery patients [15–18]. Most people who have experienced a stroke have decreased salivary function, compounded by the need to take medications that contribute to xerostomia (dry mouth) [4, 8]. Although xerostomia increases discomfort and pain particularly during eating, it increases the risk of oral-mucosal infections, tooth decay and loose teeth [8, 19]. Maintaining quality oral hygiene is a key preventive intervention for aspiration pneumonia and xerostomia.

Guidelines across the world have highlighted the need for patients to receive oral care after a stroke, yet there is insufficient evidence to determine the protocols and models of care that should be implemented to ensure comprehensive and patient-centred care [20]. In both acute and rehabilitation settings, patients may require frontline staff to assist in the provision of their oral health [7]. Although a scoping review has highlighted nurses and other allied health clinicians are ideally positioned to support patients’ oral health needs [21], qualitative findings across both Australia and the United Kingdom suggest the need for formal training in providing oral care and assessments for nurses across stroke settings [6, 22]. Based on this evidence, there is a need for an integrated dental care after stroke (IDEAS) model of care [21], involving the training of nurses and other allied health clinicians in the promotion of oral health care and early identification of oral health problems after stroke. Furthermore, a referral pathway should be established as part of this model, whereby patients with identified oral health problems could receive treatment from dental professionals. It is hoped that such a model could help to reduce the incidence of oral health-related complications such as aspiration pneumonia among stroke patients. However, this model would need to meet the needs of stroke patients to be successful.

Two studies in the United Kingdom [6, 23], identified that while stroke patients recognised the importance of oral health for their overall health, assistance with managing their oral health tended to be poor, especially in the acute care ward. Patients highlighted that some nurses made assumptions on who could manage their own oral health, were too busy or were not expected to provide oral care, and that there was a lack of dental products and information at the hospital [6]. However, the oral care needs of stroke patients in both acute and rehabilitation settings in Australia is not known, where hospital policies and training for some staff may differ in context. To understand how to improve the delivery of oral care in Australia to patients following a stroke, there is the need to explore stroke patients’ knowledge, attitudes, practices relating to oral health, and their perceptions of an integrated dental care after stroke (IDEAS) model.

## Methods

### Study aims

This study aimed to understand the perceptions about oral health for stroke survivors in both acute and rehabilitation settings.

The specific objectives were:

1. to understand the oral health knowledge, attitudes and practices of stroke patients, and
2. to understand the opinion of stroke patients about the IDEAS program.

### Study design

A descriptive qualitative approach was used to examine the perceptions of patients who had recently experienced a stroke event.

### Participant and Sampling Strategy

The study was conducted at two metropolitan hospitals in Sydney, Australia, where the target population consisted of stroke patients. Using a purposive sampling technique, patients were recruited by a nursing unit manager (NUM) from the acute stroke unit at one hospital, and from the rehabilitation ward in another hospital. In the acute hospital, participants were approached for the study once they were stabilised and within the first 7 days after admission to the unit. They were approached if they had a modified Rankin Scale (mRS) of 0–4 [24] and were not dependent on a carer. Carers of two participants were also present during the interview for additional support but did not participate in the interview. Patients participated in face-to-face semi-structured interviews conducted at the bedside in the hospital, at a time convenient for each participant.

### Data collection

All interviews were conducted by the lead researcher (SA), who is an experienced dentist and senior researcher in oral health promotion and research. The interviewer did not have any prior relationship with participants. The NUM approached the participants to inform them about the study and provided the information sheet. They were given time to decide if they wanted to participate. Once they gave their verbal consent to the NUM, the researcher organised a time to conduct the interview. Prior to each interview, the interviewer informed patients regarding the study and what participation involved, providing the opportunity for participants to ask any questions relating to the study before obtaining informed consent to participate. The investigator used a brief schedule to guide interviews [23, 25] (Additional file 1). The brief schedule was developed in consultation with an interdisciplinary expert panel and was used as a guide since the interviews were semi-structured. Interviews were conducted until data saturation was reached. All interviews were audio-recorded, with interviews lasting approximately 30 min to one hour.

### Data analysis

Audio recordings were professionally transcribed verbatim, and de-identified prior to analysis. A framework analysis was employed to analyse the data [26]. NVivo software was used to assist with coding. After reading each transcript for familiarisation, the data was reviewed, coded and indexed based on a thematic framework arising from a priori issues. To improve credibility and trustworthiness, triangulation was employed in analysing the transcripts by relating categories ‘horizontally’ in the context in which they were used. Similarities and differences between the two sites were also explored. A peer-checking process was also undertaken to refine the codes and themes. One study investigator (ACK) coded the transcripts into the framework. The principal investigator (SA) then reviewed and provided feedback on these codes. Based on this feedback, a third investigator (ARV) refined the initial codes to further develop the themes and associations, and map the framework.

## Results

Eleven stroke patients from the two hospitals admitted to an acute ward (Site 1 [S1], n = 5) or rehabilitation unit (Site 2 [S2], n = 6) participated in the study, after which the data reached saturation. Most of the participants were male (n = 8) and the mean age was 59 years. All patients who were approached for the study provided informed consent. The paper presents their perception about oral care post stroke.

### Findings

Three major themes were produced (Table 1).

**Table 1:** Major themes and sub-themes

Theme	Sub-theme
Perceptions around oral health	Attitudes
	Awareness
	Oral health status
	Oral hygiene practices
Accessing dental services	Barriers
	Facilitators
Patient recommendations for oral care	Role of nurses
	Integration of care

### Perceptions around oral health

#### Attitudes

The majority of patients considered their oral health to be critically important to their health. Some participants recognised the need to maintain a *“clean and hygienic”*[S1_2] standard of oral care and highlighted the importance of dental hygiene.

I like to make sure my teeth are clean so I don't get any gum disease [S2_7]

because teeth become quite expensive [S2_5]

Others stressed that maintaining dental hygiene was imperative, and this was perceived as part of their personal self-care.

You have to, you know. You have to look after you shave, you look after your face and everything, you’ve got to look after your teeth [S1_2].

#### Awareness

Only two participants recognised the impact of poor oral health on their systemic health, describing the relationship between oral health and diabetes and nutrition:

[I’ve had] poor oral health and, the diabetes doesn’t help. [S1_5]

It’s very important because if we have good teeth and a good mouth, you can eat properly and you can chew your stuff up soft and that, so you won’t have problems with your stomach as well. [S2_2]

Further, few were aware of the importance of oral health care following stroke and its relationship with aspiration pneumonia, particularly in the context of dysphagia.

if they are bed ridden, that’s of course a huge problem [S1_4]

well, people die from it [S1_3]

Only one participant had some knowledge about the pathophysiology of stroke, commenting that they had some *“knowledge about the brain”*[S1_4]. All other participants could not identify how stroke could impact their oral health.

in stroke and oral care I don’t know what the relationship is, not at all? [S2_7]

Some misconceptions around appropriate oral health interventions also emerged. Another participant considered poor oral health to be inevitable.

somebody gets a bit of a toothache they [can] just take a couple of Panadols or something and hope it goes away [S2_7]

Yes, it’s impossible to look after my teeth…old age is catching up and the tooth is getting rotten and losing my tooth…I will end up with no tooth [sic] one day [S2_2]

#### Oral health status

More than half of participants reported experiencing oral health problems. These problems included missing teeth, pain, loose teeth, difficulties in chewing, dental decay, broken or chipped teeth, and receding, tender or bleeding gums.

Well I always been having an infection down the root and they’ve been drilling down the root and put antibiotic, all that stuff to ease the pain…it’s getting rotten now [S2_2]

Last year I had all the dental work done yeah all up about one, two, three, five crowns…my gums got a bit sore and tender and they were receding… [S2_7]

#### Oral hygiene practices

Almost three-quarters of participants reported regularly brushing their teeth at least once a day. Three people reported flossing or used inter-dental products, and one person used a mouth wash, stating, “Oh I brush my teeth every morning…And I get back to brushing my teeth every night…and I floss after meals.”[S2_7].

A number of participants also reported using a manual toothbrush with toothpaste over an electric toothbrush. A few participants reported that their current dental self-care was “mostly” or “still the same” as it was pre-stroke.

Three participants reflected that their oral care had deteriorated since their stroke. They attributed this to weakened motor control and changes to facial/mouth sensitivity.

I find that when you’re one handed it’s very difficult – […] and, most people that have suffered a stroke do tend to lose control… [S1_5]

When I brush—my tongue, I can’t brush it ‘cause [vomiting sounds]… um, before the stroke I can brush it quite [well]. [S2_6]

Only one participant observed that their oral health care increased after their stroke.

I have one working arm and, it happens to be the one that’s good. So, I’m left handed – I can brush my teeth like this…with the toothbrush and, put toothpaste on the brush and, I do the rest… [S1_5].

Most participants reported that they were offered with a toothbrush and toothpaste during their hospitalisation; however, the level of dental assistance and care provided by clinical nurses varied, even within the same hospital. Only three participants reported some assisted dental care while all other participants reported that they received no assistance during admission or received any information about their oral health.

Ah – they just ask you, have you cleaned your teeth, have you showered, blah, blah blah. They ask the question. [S1_3]

No, I don’t think anybody is [looking after my teeth] [S1_2]

Only two participants were asked about their dentures. Two participants from the same site (S2), one of which had dentures, were assessed for their ability to swallow and any weakness in their facial muscles. No participant could recall having been assessed for their oral health when they were admitted into hospital.

### Accessing dental services

#### Barriers

Almost all participants identified a combination of factors that deterred their decision access to dental care services. Some participants disclosed that although they saw the value in accessing the dental service and did visit the dentist, they would visit the dentist infrequently due to cost.

What stops me from seeing the dentist all the time is that I can’t afford it. [S2_2]

Among those who cited cost as a barrier, there was also a lack of information provided to patients about accessing public dental services.

I can always go to the dentist, but…I don’t know where the public dentists are…because nobody’s giving any information yet, even my family doctor [S2_2]

A number of participants identified that time constraints was “one of the biggest barriers” [S1_5] that limited their access to the dentist. For these participants, stroke had affected their ability to drive, restricting their transportation options.

I’m not driving at the moment; I don’t know when I’m going to be driving… [S2_1]

One participant, who required carer support post-stroke, identified the paucity of patient transport options.

…they said you have to come to the dental hospital. I said I can’t come by myself - you have to … give me some, some transport to come. They said, we don't have…I said there is no transport I can’t come because I have to book for my - my carers to come and pay the carers - and I cancelled it [S2_8].

Other barriers to accessing dental services included poor attitudes towards oral health, difficulty in locating a reputable dental practitioner, long waiting times and age.

Or maybe they just can’t be bothered or too lazy… [S2_7]

…I don’t know [which dental practitioners are] good. [S2_6]

Sometimes my dentist doesn’t have time to see me I have to wait for one month sometimes. [S2_8]

#### Facilitators

**Fig. 1:**
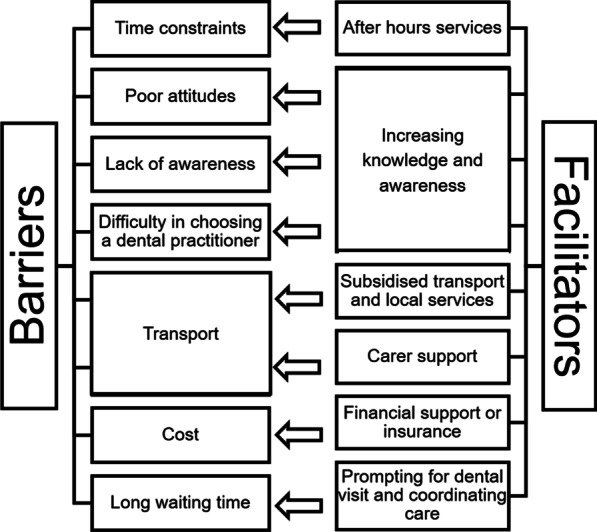
Barriers and facilitators to access dental services

Participants suggested a range of strategies that would improve their access to dental services (Fig. 1). These included additional financial support, prompting.

for dental visits and assistance in coordinating care, subsidising transport and local services, carer support, increasing oral health knowledge and awareness, and after-hours services.

One participant agreed that additional financial *“support of course would probably* [encourage people]*…if they can’t afford” [S*1_4] to access dental services more regularly. The two participants who had private health insurance reported that it mitigated their dental costs, explaining that cost was not a factor *“because I’ve been in* [a private health fund] *all my life” [S*1_1].

Two participants agreed that more coordination of care with their dental provider to arrange appointments *“would work” *[S2_1] to improve access to dental care. They specified that this included having scheduled appointments and reminders.

They always – um – call every six months and…They schedule my meeting or my appointment and, then I – I just turn up. And, they also…schedule it in the smart phone or you have a text – you have a text – text message. So it’s pop [sic] up. [S1_4]

Participants also cited that the availability of subsidised transport in the absence of a carer and the availability of services that were local and open after business hours would also facilitate access.

Before I had a car I used to drive a vehicle, now maybe subsidy taxi. [S2_8]

### Patient recommendations for oral health care

#### Role of nurses

Participants agreed that nurses were well-placed to assist with oral health care in both acute and rehabilitation settings. Most also agreed that nurses were qualified to provide oral health education and conduct a dental assessment, depending on whether they had previously received specific oral health training or experience.

If they’ve got more experience about that, the nurses would be okay; it would be a good idea [S2_2]

It’s [training program] gonna make a difference and, it would be appreciated by the patients [S1_3]

However, one participant explained providing dental care may not be feasible because nurses have “got a bit – too much on their plate to worry specifically about your teeth”[S2_6].

#### Integration of care

Participants suggested that nurses could integrate oral health care by reminding patients during their checks and upon admission. The majority of participants preferred to receive oral health information through brochures, verbally or both.

Most people are aware of their need for oral hygiene but it wouldn’t hurt to be a bit of a reminder when you come in, are you looking after your teeth, are you brushing your teeth… [S2_7].

Paper sheets and…nurses and staff can take some initiative to help that patient and give some knowledge. [S1_2]

There was also general agreement that they were likely to visit the dentist if a referral pathway was established. Others highlighted that an integrated dental care model where nurses could assess patients for dental problems, arrange an appointment, and refer patients to the dental service would “take everything out of the equation and actually [put] it on a plate for you” [S1_1].

Although some participants agreed on having a referral pathway established, they preferred receiving an oral health assessment by a dental professional due to their knowledge and experience.

[a dental professional could] have a look at it and fix what the problem is and then they’ll tell me what to do and I’ll carry on looking after me [S 2_2]

## Discussion

The findings from this study have indicated that current models of care that seek to provide comprehensive oral healthcare for patients in the context of stroke are failing to meet the needs of patients.

### Perceptions around oral health

Although many of the participants were aware of the importance of maintaining good oral health they did not recognise the impact of poor oral health on their general health. Among the study participants many reported having a range of dental and gum problems that were not addressed. This is a complex problem that requires a collaborative effort from all healthcare professionals in the multidisciplinary team and partnership with patients and families. Patients are most vulnerable of oral health deterioration after stroke [27]; although patients in this sample perceived oral health to be important, their fundamental oral care needs were largely unmet. Globally, oral health is at *‘tipping point’* and there is need for new integrated approaches. It is recognised that the global burden of oral health cannot be addressed by dentistry and oral health practitioners alone in silo, but requires an integrated oral health approach, where other disciplines play a critical role in improving oral health outcomes [28]. This is critical in the specialty of stroke where patients experience considerable disadvantage and comorbidities post stroke.

Whilst international, national and local clinical practice guidelines for stroke exist, our study findings echo that implementation of guideline recommendations remains poor. In Australia, the bi-annual acute stroke audit exists, yet there is limited scope to benchmark on this element of care. Further, there are limited incentives in place to encourage better implementation of evidence-based guidelines. Whilst guidelines are critical to guide practice, key barriers to implementing guidelines may include a lack of valid, reliable and easily implementable oral health assessment tools [29]. A relatively new Comprehensive Oral Assessment Tool for Stroke Patients (COATS) has been developed. The tool was validated among registered and unregistered nurses in the UK [6]. The instrument examines patient’s ability, oral cleanliness and oral comfort. There is scope to increase the uptake of such tools into routine clinical practice.

### Accessing dental care

Access to dental treatment can be difficult for those with a disability. In addition to restricted accessibility to attend dental services due to physical limitations, there are wider psychological, sociological, legal and economic factors that often act as barriers [30]. Lack of affordability is often perceived to be the main barrier. Financial barriers include the direct cost of dental care and indirect costs like cost of transportation and cost to the carer. Many may also have difficulty obtaining information regarding available services and managing dental appointments. Participants in our study had very similar barriers. Access to dental care appeared problematic due to cost, ability to make appointments, lack of awareness of services and importance, lack of mobility and transport. Key recommendations based on patients’ suggestions to improve oral health services included nurses assisting in the hospital setting, reminders and information about initiative about oral health and models or care or referral pathways to access dental services.

### Patient recommendations for oral care

Our previous research looking at the nursing and allied health perspectives of quality oral care after stroke noted that most staff felt they did not have adequate knowledge, resources and training to administer oral care in the hospital setting [22]. Routine oral healthcare education was not provided to patients. Yet promotion of oral health was viewed as a fundamental aspect of care. A large-scale US survey conducted by Carson et al. [31] of nurses working in adult ICU units identified that only 56% of ICU nurses reported that their employer had written oral hygiene protocols for patients receiving mechanical ventilation. Sixty-five percent of respondents reported that they brushed patients’ teeth at least every 12 h; however, 33% reported that they either brushed patient’s teeth only as needed or rarely/not at all [31]. Similar research by Sanchez et al. [32] identified that among people with cardiovascular disease, oral care was only initiated if prompted by the patient, if they had diabetes, or was part of the pre-admission surgical checklist. In the present study, most participants believed that nurses played an important role in providing oral care in both the acute and rehabilitation setting. The participants suggested to integrate oral health care in general assessment checks at the time of admission to embed routine oral care during the stay. Although the role of stroke clinicians promoting oral health in hospitals is not explored in Australia, the literature supports a positive role of nursing staff in oral health across other settings such as aged care [33].

### Strengths and limitations

Our study addressed an important gap in the current stroke literature. This study presents a real worldview of patients’ perceptions and oral healthcare needs post-stroke in Australia. This study targeted relatively functional stroke patients, with a mRS of 0–4, and not dependent on a carer. However, this study is not without its limitations. Our sample size was relatively small as many patients in the acute facility were unable to consent for the interviews. Furthermore, many who participated had some difficulty with verbal communication with the interviewer, which may impact quality of data collected. Some patients may have felt obliged to participate as the NUM was involved in recruitment; however, it was emphasised that participation was voluntary and non-participation would not affect quality of patient care. Since interviews were conducted at bedside, some participants may have provided responses that were less open. In addition, the findings are from two settings, and there is a potential for recall bias.

## Conclusions

This study has shown that despite recognising the importance of oral health, stroke survivors are experiencing dental problems and have limited awareness of its impact on general health. Further, there is variability in their oral hygiene practices and few are receiving oral health care assistance from nurses or access dental services. Although no integrated oral health care models currently exist for stroke patients, there is scope for non-dental health professionals to contribute to a new collaborative, integrated model of oral healthcare for individuals after stroke, such as the IDEAS program proposed in this paper. These models should build the capacity of non-dental professionals in oral health care and early recognition of oral health problems. Future research should focus on the design and implementation of such integrated models of care.

## Supplementary Information

**Additional file 1. Interview guide with patients.**

## Data Availability

The datasets analysed may be available from the corresponding author on reasonable request.

## Abbreviations

COATS: Comprehensive Oral Assessment Tool for Stroke Patients
IDEAS: Integrated dental care after stroke
mRS: Modified Rankin Scale
S1: Site 1
S2: Site 2
