# Supplementary material for: Patient perceptions of oral health care following stroke: a qualitative study
Source: BMC Oral Health. 2021 Mar 17;21:127. doi: 10.1186/s12903-021-01501-7 (PMC7968245; doi:10.1186/s12903-021-01501-7)
Supplement: Supplementary file 1 — Additional file 1. Interview guide with patients. [file 12903_2021_1501_MOESM1_ESM.pdf]

Additional File 1 – Interview guide with patients

- Oral health habits prior to stroke and their motivation to return to that level of care
- Their perception of the importance of oral health
- Knowledge about stroke and its effect on oral health, including their understanding of oral and chest infections and pneumonia
- How they access oral healthcare (frequency and type of care)
- Barriers and facilitators to accessing dental care
- How they would prefer oral health administered to them?
